# Genome-wide association and biparental mapping revealed a major quantitative trait locus associated with seedling resistance to bacterial leaf streak in durum

**DOI:** 10.1007/s00122-025-05111-7

**Published:** 2025-12-19

**Authors:** Fazal Manan, Xuehui Li, Agnes Szabo-Hever, Gongjun Shi, Elias Elias, Justin Faris, Steven S. Xu, Zhaohui Liu

**Affiliations:** 1 Department of Plant Pathology, North Dakota State University, Fargo, ND 58102 USA; 2 Department of Plant Sciences, North Dakota State University, Fargo, ND 58102 USA; 3 USDA-ARS Cereal Crops Improvement Research Unit, Edward T. Schafer Agricultural Research Center, Fargo, ND 58102 USA; 4 USDA-ARS, Crop Improvement and Genetics Research Unit, Western Regional Research Center, Albany, CA 94710 USA

## Abstract

**Key message:**

We identified sources of resistance and a major QTL for resistance to bacterial leaf streak in durum.

**Abstract:**

Bacterial leaf streak (BLS), caused by *Xanthomonas translucens* pv. *undulosa*, has reemerged as a significant disease impacting bread wheat and durum production worldwide. The lack of resistance sources and limited understanding of the genetics behind host resistance have made breeding for BLS resistance challenging, especially in durum wheat. In this study, we first evaluated the reaction of a set of durum cultivars, mainly from North Dakota State University (NDSU). Our results indicated that most cultivars were susceptible, with only a few exceptions. Next, we utilized a subset of the Global Durum Panel (GDP) to identify sources of resistance and conduct a genome-wide association study (GWAS) to identify BLS resistance loci. Additionally, we performed disease evaluations and quantitative trait locus (QTL) analysis on two durum recombinant inbred line (RIL) populations: one was derived from the resistant NDSU cultivar ‘Ben’ and the other from the resistant Ethiopian landrace PI 387336. Our findings revealed that several modern durum cultivars from different countries within the GDP exhibited a high level of resistance. Both GWAS and biparental mapping identified a major QTL located at the distal end of chromosome arm 6AS. The physical positions of the single-nucleotide polymorphism (SNP) markers obtained from both experiments strongly suggest that the same locus is responsible for BLS resistance in the tested durum materials. The resistant accessions and SNP markers identified in this study will be valuable for transferring this major QTL into elite durum and common wheat cultivars.

**Supplementary Information:**

The online version contains supplementary material available at 10.1007/s00122-025-05111-7.

## Introduction

Bacterial leaf streak (BLS), also known as black chaff and caused by *Xanthomonas translucens* pv. *undulosa* (Xtu), is a widespread disease affecting wheat, including common wheat (*Triticum aestivum* L., 2*n* = 6*x* = 42, AABBDD genomes) and durum (*T. turgidum* L. ssp. *durum* (Desf.), 2*n* = 4*x* = 28, AABB genomes). This disease was observed in many places around the world in the late nineteenth century, but the causal bacterium was not formally identified and described in the USA until 1919 (Smith et al. 1919). BLS has been formally reported in many countries and is considered a global concern (Duveiller et al. 1997; Sapkota et al. 2020; Ledman et al. 2023). In the USA, BLS has traditionally occurred sporadically in warm and humid areas, particularly in the South (Duveiller et al. 1997; Milus and Mirlohi 1995). However, over the past two decades, outbreaks have become more frequent in the Upper Midwest region, where most spring wheat and durum wheat is grown (Adhikari et al. 2012a; Kandel et al. 2012; Curland et al. 2020). BLS can lead to significant yield losses, with reports indicating reductions of up to 40% (Waldron 1929; Forster and Schaad 1988; Shane et al. 1987; Duveiller and Maraite 1993; Tillman et al. 1999). Recently, Friskop et al. (2023) found that highly susceptible hard red spring wheat cultivars in North Dakota experienced a staggering 60% yield loss due to BLS. The economic impact of this disease in North Dakota was estimated at $4.7 million in 2019 and $8.0 million in 2020 (Friskop et al. 2023).

Chemical methods are ineffective for controlling BLS in the field, making genetic resistance the only viable option. Disease evaluations and screenings have been conducted on common wheat germplasm due to its significant economic importance worldwide. Those studies have included a variety of materials such as cultivars, breeding lines, landraces, and other related specimens (Hagborg 1974; Akhtar and Aslam 1986; Duveiller et al. 1993; Alizadeh et al. 1994; Milus and Mirlohi 1994; El Attari et al. 1996; Milus et al. 1996; Tillman et al. 1996; Adhikari et al. 2011; Kandel et al. 2012; Sapkota et al. 2018; Ramakrishna et al. 2019; Acharya et al. 2024). While complete resistance has not been identified, several wheat genotypes exhibit partial resistance, with some demonstrating high levels of resistance. For instance, ‘Boost,’ a hard spring wheat cultivar, has been recognized as one of the most resistant spring wheat lines (Acharya et al. 2024). Additionally, several triticale accessions have also shown high levels of resistance (Sapkota et al. 2018).

Genetic analysis and mapping of BLS resistance have been conducted in common wheat. Duveiller et al. (1993) identified five loci (*Bls1, Bls2, Bls3, Bls4*, and *Bls5*) in winter wheat that confer resistance to BLS. Using genome-wide association mapping, Adhikari et al. (2012b) found five novel genomic regions associated with BLS resistance located on chromosomes 1A, 4A, 4B, 6B, and 7D in spring wheat landraces. Kandel et al. (2015) applied identity-by-descent mapping and identified BLS resistance quantitative trait loci (QTL) on chromosomes 2A and 6B in a spring wheat line. Additionally, Ramakrishnan et al. (2019) reported five genomic regions on the chromosome arms 1AL, 1BS, 3AL, 4AL, and 7AS associated with BLS resistance by conducting a GWAS on a winter wheat panel. More recently, Acharya et al. (2024) identified QTLs on chromosomes 3B, 5A, 5B, and 7D in the spring wheat cultivar ‘Boost’ and the synthetic wheat accession M6 associated with BLS resistance. While most studies indicate that BLS resistance is polygenic in common wheat, Wen et al. (2018) reported the identification of a single dominant resistance gene, *Xct1*, located on 5R in triticale, which confers resistance to the wheat BLS pathogen.

Durum wheat represents approximately 7% of global wheat production, with an annual output of around 35 to 40 million metric tons (MMT) in the last decade (Broccanello et al. 2023). It is primarily cultivated in West Asia, North and East Africa, the North American Great Plains, India, and certain regions of Eastern and Mediterranean Europe (Peters-Haugrud et al. 2025). Over the past five years, the USA has averaged a production of 1.5 MMT of durum wheat (U.S. Wheat Associates, https://uswheat.org/crop-and-quality/durum, accessed June 2025). North Dakota and Montana are the two major producers, accounting for 90% of the total durum production in the USA (U.S. Wheat Associates, https://uswheat.org/crop-and-quality/durum, accessed June 2025). Epidemics of BLS in wheat have been reported in North Dakota and surrounding areas. However, the response of current durum cultivars in North Dakota remains unknown, and little has been done to screen durum germplasm for BLS resistance or to map the genetic loci associated with this resistance. One recent study reported the screening and GWAS for BLS resistance in cultivated emmer wheat (*T. turgidum* ssp. *dicoccum*), which is a close relative of durum wheat (Khan et al. 2025). The study found that a very low percentage of accessions was resistant and a few markers on 1AS was associated with resistance.

Because so little work has been done to identify sources of BLS resistance and resistance genes/loci for durum wheat improvement, the objectives of this work were to: (1) determine the reaction of various durum cultivars from the North Dakota State University (NDSU) durum breeding program; (2) identify sources of resistance to BLS by evaluating a subset of durum accessions from the Global Durum Panel (GDP) and determine the genomic regions associated with resistance using GWAS; and 3) confirm quantitative trait loci (QTL) that confer BLS resistance in two durum biparental populations.

## Materials and methods

### Plant materials

Three sets of materials were used in this study. The first set included a total of 14 durum cultivars released from 1952 to 2017 by the NDSU durum breeding program (Table 1). The set of materials was used to gain information on the reactions of the ND durum cultivars to BLS. The second set of materials consisted of 511 durum accessions (Table S1), a subset of the Global Durum wheat Panel (GDP) (Mazzucotelli et al. 2020). The subset consisted of 410 modern cultivars or breeding lines, 36 landraces, and 65 EPO (Evolutionary Pre-breeding pOpulation) lines from 14 countries or international breeding centers (Table S1). The third set of materials were two durum recombinant inbred line (RIL) populations, designated as BP025 and RP336. The BP025 population consisted of 200 RILs developed from the cross between the NDSU durum cultivar ‘Ben’ (PI 596557, Elias and Miller 1998) and the cultivated emmer wheat (*T. turgidum* ssp. *dicoccum* (Schrank ex Schübl.) Thell.) accession PI 41025 (Faris et al. 2014). The RP336 population consists of 198 RILs from the cross between NDSU durum line Rusty and the Ethiopian durum landrace PI 387336 (Liu et al. 2020). The hard red spring wheat cultivars Boost (resistant) and RB07 (susceptible) and the triticale cultivars/lines 8A-312 (PI 428854, resistant) and UC38 (Clxt 31, susceptible) were used as resistant and susceptible checks in all greenhouse disease evaluations (Table 1, Acharya et al. 2024).

**Table 1:** Reaction of durum cultivars from North Dakota State University to bacterial leaf streak

Cultivars and lines	Type^a^	PI No	Year released	AvIT^b^	Av%WS^c^
8A-312	Triticale	PI 428854	1976	1.0f	2.0f
Boost	Common wheat	PI 678681	2015	1.1f	6.5ef
UC38	Triticale	Clxt 31	1973	4.3a	68.3a
RB07	Common wheat	PI 652930	2007	4.5a	65.0a
Rusty	Durum wheat	PI 639869	2004	3.9ab	63.3a
Lebsock	Durum wheat	PI 613620	1999	3.5bc	41.6bc
Langdon	Durum wheat	CItr13165	1956	3.5 cd	48.3b
Caprio	Durum wheat	PI 670039	2012	3.4 cd	45.0bc
Tioga	Durum wheat	PI 660664	2010	3.3 cd	35.0c
ND Grano	Durum wheat	PI 687795	2017	3.8ab	40.1c
Grenora	Durum wheat	PI 642022	2005	3.3 cd	36.6c
Dilse	Durum wheat	PI 632367	2002	3.2 cd	35.0c
Mountrail	Durum wheat	PI 607530	1998	3.1d	46.6c
ND Riveland	Durum wheat	PI 687796	2017	3.0d	30.4c
Divide	Durum wheat	PI 642021	2005	2.0e	19.1d
Alkabo	Durum wheat	PI 642020	2005	2.0e	18.7d
Pierce	Durum wheat	PI 632366	2001	2.0e	15.1de
Ben	Durum wheat	PI 596557	1996	1.8e	14.1de

^a^Two common wheat and two triticale lines were included as resistant and susceptible checks

^b^AvIT = average infection type (IT). Infection type for each cultivar was recorded based on a 0–5 scale (Achary et al. 2024) with 0 being immune and 5 being highly susceptible. The same letter (s) indicates no significance difference based on Tukey’s HSD test

^c^Av%WS = average percentage of water-soaked area (%WS) was used to estimate the infected leaf area ranging from 0 to 100. The same letter indicates no significance difference based on Tukey’s HSD test

### Disease evaluations in greenhouse

Disease evaluations were separately conducted for three sets of materials. For each set of materials, disease evaluations were conducted in three experiments with each experiment containing two replications arranged in a completely randomized design. To prepare plants for evaluations, two seeds of each line were planted in a small cone (4 cm diameter × 13 cm deep, Stuewe & Sons, Inc., Corvallis, OR USA) filled with Sunshine SB100 soil (SunGrow Horticulture, Bellevue, WA, USA). A small amount of ‘Osmocote Plus’ 15–19-12 fertilizer (Scotts Sierra Horticultural Product Company, Maysville, OH, USA) was uniformly applied to all plants at planting. All the cones were placed in 98RL racks with the border cones planted with the susceptible check RB07. The plants were inoculated at the two-to-three leaf stage in the greenhouse (about 14 days) as described below.

The inoculum was prepared following the protocol described by Wen et al. (2018). Briefly, the highly virulent Xtu strain BLS_P3 was grown on Wilbrink’s agar (WBA) for two days at 28 °C. The bacterial cells were harvested from the culturing plates and suspended in 1 × PBS buffer (8 g NaCl, 200 mg KCl, 1.44 g Na_2_HPO_4_, 2.45 g KH_2_PO_4_, 1L of distilled water, and pH = 7.4). The final inoculum concentration was adjusted to OD600 = 0.5 (1 × 10^8^ CFU/ml) for spray inoculation. Tween-20 (polyoxyethylene sorbitan monolaurate, Sigma-Aldrich) was added to the final bacterial inoculum at one drop per 100 mL.

The inoculation was done by direct spraying with a spray gun connected to an air pump having a setting of 30–40 psi for air pressure. The plants were sprayed until the liquid inoculum solution began to run off the leaves. The inoculated plants were placed in a misting chamber for two days at room temperature under 14 h photoperiod and 100% humidity. Then, the plants were kept for two days in a plastic tent (set in the greenhouse room) that had a temperature range of 25–30 °C under 14 h photoperiod and approximately 70% humidity. Disease severity was scored 7 days post-inoculation using both an infection type (IT)-based scale and the percentage of water-soaking area (%WS) developed on the secondary leaves as described in Acharya et al. (2024). The IT scale ranged from immune (0.0) to highly susceptible (5.0) and %WS ranged from 0 to 100.

### Statistical analysis

Data normality and homogeneity of variances across replications were tested using SAS 9.4 (SAS Institute, Cary, NC, USA). Data normality was assessed using the Shapiro–Wilk test (Shapiro 1965) while homogeneity of variances across replications was assessed by using Levene’s test (Levene 1960). One-way analysis of variance (ANOVA) and the Tukey’s honestly significant difference (HSD) test was performed in SAS to determine significant differences in the disease scores among durum cultivars and accessions at *α* = 0.05. Pearson correlation (Benesty et al. 2009) was performed to test the correlation between infection type and percent water-soaking disease scoring. The infection-type and %WS data from homogenous replications were combined and the average data across replications, designated AvIT and Av%WS, were used in the subsequent GWAS and QTL mapping.

### Genome-wide association mapping

All accessions in the GDP were previously genotyped using the Illumina iSelect 90 K single-nucleotide polymorphism (SNP) array at the USDA-ARS Small Grains Genotyping Laboratory, Fargo, ND (Wang et al. 2014; Mazzucotelli et al. 2020). The original marker data of the GDP (13,373 SNPs) were downloaded from the GrainGenes website (https://wheat.pw.usda.gov/GG3/global_durum_genomic_resources). The marker data for the 511 genotypes used in disease evaluation were extracted from the dataset and filtered by applying minor allele frequency (MAF) > 5% and no more than 10% missing data for a given markers using Tassel V5.0 tools (https://www.maizegenetics.net/tassel).

A total of 13,072 SNP markers were obtained and used to conduct a principal component analysis and a centered kinship (K) matrix in TASSEL (Bradbury et al. 2007). Based on the scree plot (Fig. S1), the first seven principal components (PCs) were selected for model-based cluster analysis, which was performed using the R package Mclust (Scrucca et al. 2023).

Marker-trait association analysis was performed using TASSEL 5.0 (Bradbury et al. 2007). Four statistical models were tested, and they included: (1) simple association analysis using a general linear model (simple model); (2) a general linear model including the first seven PCs as covariates (P model); (3) a linear mixed model including kinship matrix (K model); and (4) a linear mixed model including population structure and kinship matrix (PK model). The mean of the squared difference (MSD) between observed and expected p values was calculated for each model. The best model for each trait was determined as the model returning the smallest MSD value. The false discovery rate (FDR) was calculated from p values using the R function p.adjust (method = FDR) (Benjamini and Hochberg 1995). Significance of marker-trait association is defined by FDR as a q value smaller than 0.05 [-Log10 (0.05) = 1.3]. Multiple loci mixed model (MLMM) and BLINK (Bayesian-information and Linkage-disequilibrium Iteratively Nested Keyway) models implemented in Genomic Association and Prediction Integrated Tool (GAPIT) were also used to detect significant markers associated with traits as described in Wang et al. (2022).

### Genetic linkage maps and QTL analysis

The genetic linkage maps of both durum populations BP025 and RP336 have been published previously (Faris et al. 2014; Liu et al. 2020). The genetic linkage map of BP025 consisted of a total of 2,593 polymorphic markers (SNP and SSR) and covered all 14 chromosomes with a total distance of 2,444.4 cM (Faris et al. 2014). The genetic linkage map of RP336 consisted of a total of 2,894 SNP markers (from genotyping by sequencing) spanning 2,858 cM in genetic distance covering all chromosomes (Liu et al. 2020). The two populations have been used previously to identify QTL associated with agronomic and domestication traits and resistance to tan spot and Hessian fly (Faris et al. 2014; Liu et al. 2020; Guo et al. 2020; Peters-Haugrud et al. 2023; Anderson et al. 2025). The previously published marker and linkage map data was directly used with the obtained phenotypic data in the QTL analysis by using QGene v.4.3 software (Joehanes and Nelson 2008). Simple interval mapping was used to locate genomic regions associated with BLS resistance and estimate phenotypic variation for each QTL. Composite interval mapping (CIM) was used to refine the QTL region by selecting default co-factors. Permutation tests with 1,000 iterations determined the Logarithm of Odds (LOD) thresholds of 3.2 and 4.7 for SIM and CIM, respectively to detect significant QTL.

## Results

### Reaction of durum cultivars to BLS

There were significant differences in the reaction to BLS among durum cultivars and four checks as measured by IT and %WS (*F* = 21.9 and 14.5, *p* < 0.0001, Table S2), and Tukey’s HSD test classified them into different significance groups (Table 1). The triticale line 8A-312 (resistant check) was the most resistant with an AvIT score of 1.0 and Av%WS score of 1.3%. In contrast, the triticale line UC38 (susceptible check) was highly susceptible (AvIT = 4.3, Av%WS = 68.3). As expected, the common wheat resistant check Boost had scores of 1.1 and 6.5 for AvIT and Av%WS, respectively, while the common wheat susceptible check RB07 had an AvIT of 4.5 and Av%WS of 65.0. Among durum cultivars or lines, ‘Ben,’ ‘Divide,’ ‘Pierce’ and ‘Alkabo’ demonstrated lower AvIT and Av%WS scores (from 1.0 to 2.0 for AvIT, from 14.1 to 19.1 for Av%WS), whereas all the others had average disease scores above 3.0 for IT and %WS > 30.0. Rusty (AvIT = 3.9 and Av%WS = 63.3) and ‘ND Grano’ (AvIT = 3.8 and Av%WS = 40.1) were the most susceptible durum line and cultivar (Table 1).

### Reaction of GDP durum accessions to BLS

**Fig. 1:**
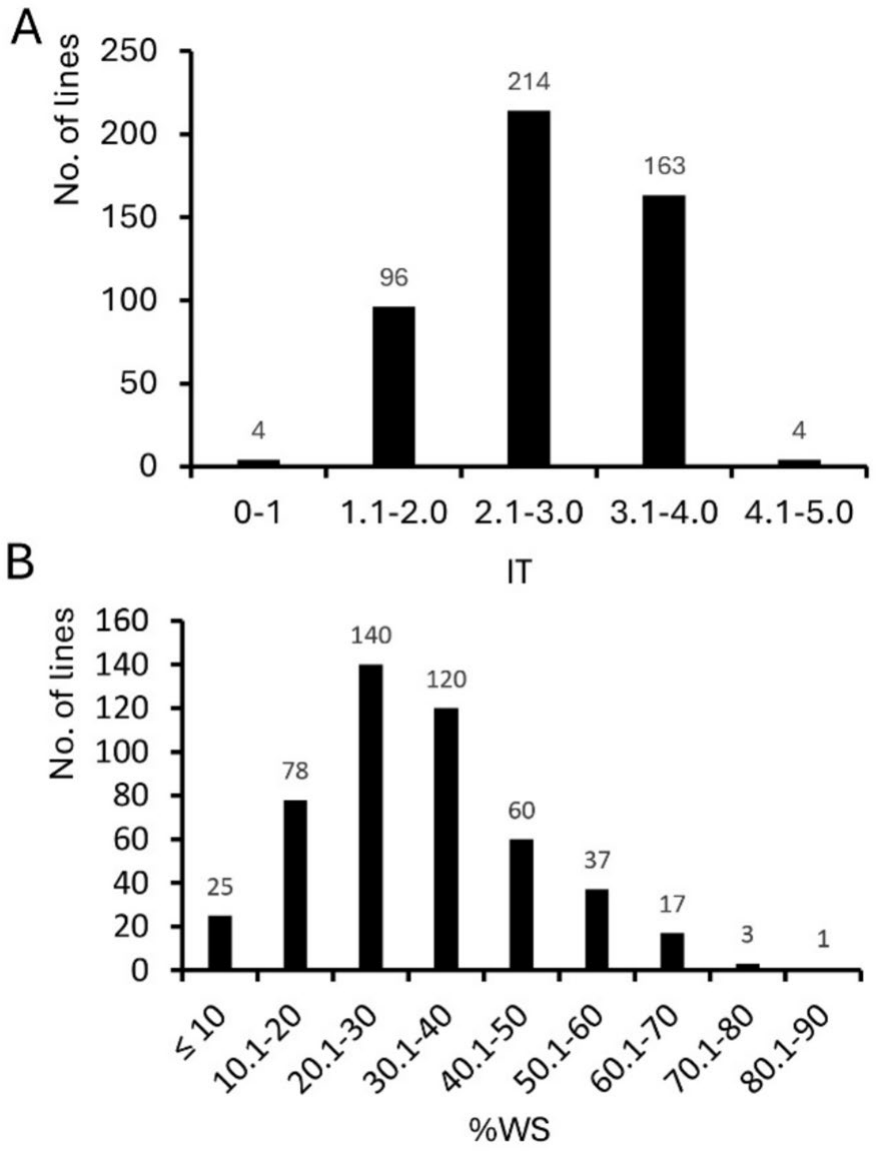
Distribution of Global Durum Panel lines for reaction to bacterial leaf streak. **A** Distribution based on average infection type (IT). Infection types were based on 0–5 scale as described in method. **B** distribution based on the average percentage of water-soaking area (Av%WS). %WS ranged from 0 to 100%

Significant differences were observed among 511 durum accessions (*F* = 4.01 for IT and 3.46 for %WS, *p* < 0.01, Table S3) in the GDP for reaction to BLS. Levene’s test of homogeneity showed that the replications of GDP accessions were homogeneous (*p* = 0.07). The disease scores of the GDP ranged from 1.0 to 4.3 for AvIT (average = 2.7) and from 2 to 83% for Av%WS (average = 33.65%) (Fig. 1A and B). Most durum accessions (79.2%) had AvIT scores greater than 2.1, suggesting susceptible or highly susceptible reactions, while the others (20.8%) had AvIT scores lower than 2.0, indicating a resistant response. Regarding Av%WS, 25 accessions scored lower than 10%, indicating a high level of resistance. The 20 most resistant durum lines from the GDP, with AvIT scores lower than 1.5 and Av%WS below 10%, are listed in Table 2. Among them, 11 were identified as cultivars, while the others were breeding or pre-breeding lines. Regarding geographic origin, nine were from France as EPO lines, five from ICARDA, two from Argentina, and one each from Spain, Australia, and Algeria. Analyzing the association among the data, a high correlation was observed between the %WS and IT data with the correlation coefficient of r = 0.88 (*p* < 0.0001).

**Table 2:** List of the most resistant durum cultivar and breeding lines from the Global Durum Panel

Line number	Accession name^a^	Improvement status^a^	Origin^a^	AvIT	Av%WS
GDPv2-025	Yerer	Variety	Ethiopia	1.3	5.7
GDPv2-046	Carilo	Variety	Argentina	1.2	4.7
GDPv2-056	CBW_08133	Breeding line	Argentina	1.5	6.0
GDPv2-065	NOBILIS	Variety	France	1.5	8.3
GDPv2-079	SENADUR	Variety	Spain	1.2	5.7
GDPv2-112	ORJAUNE	Variety	France	1.3	7.3
GDPv2-163	Ouassara1	Breeding line	ICARDA	1.2	5.5
GDPv2-194	TELSET5	Breeding line	ICARDA	1.2	6.7
GDPv2-199	Serene1	Breeding line	ICARDA	1.3	7.3
GDPv2-239	Ardente	Variety	France	1.5	9.0
GDPv2-309	Arstar	Variety	France	1.2	3.7
GDPv2-315	Floradur	Variety	Austria	1.3	8.3
GDPv2-456	EL4X_27	Pre-breeding line	France	1.0	3.7
GDPv2-458	EL4X_31	Pre-breeding line	France	1.0	3.0
GDPv2-465	EL4X_69	Pre-breeding line	France	1.2	6.3
GDPv2-474	GQ4X_91	Pre-breeding line	France	1.2	7.5
GDPv2-492	GQ4X_175	Pre-breeding line	France	1.2	7.5
GDPv2-438	Beltagy3 = AïnLehma	Variety/elite line	Algeria	1.2	3.7
GDPv2-440	IcaKader2	Variety/elite line	ICARDA	1.2	7.3
GDPv2-443	Lahnmiki	Variety/elite line	ICARDA	1.0	4.7

^a^Information was obtained from Mazzucotelli et al. (2020)

### Association mapping

PC analysis was conducted using 13,072 SNP markers for the 511 durum wheat lines, and the scree plot is shown in Fig. S1. The first two PCs explained 8.7% and 4.1% of the total variation, respectively. Model-based cluster structure analysis utilizing the first seven PCs identified seven clusters (Fig. S2; Table S4). PC1 separated Clusters 4 and 7 from Clusters 1 and 2 (Fig. S2). Within Cluster 1, 43 out of the 64 breeding lines were from ICARDA. There were 110 breeding lines within Cluster 2, where 42 were from CIMMYT and 26 were from ICARDA. Clusters 3 and 5 were more diverse and contained breeding lines from different programs and countries. Of the 16 breeding lines in Cluster 6, 11 were from ICARDA, three were from Syria, and two were from Morocco. Of the 36 landraces, 30 were grouped into Cluster 4, three were in Cluster 7, and two were in Cluster 5.

For the trait AvIT, the MSD values were 0.01714, 0.01714, 0.00063, and 0.00017 for the simple, P, K, and PK models, respectively. For the trait Av%WS, the corresponding MSD values were 0.01280, 0.01280, 0.00155, and 0.00030. Based on these values, the PK model was the best-performing model for both traits, AvIT and Av%WS.

**Fig. 2:**
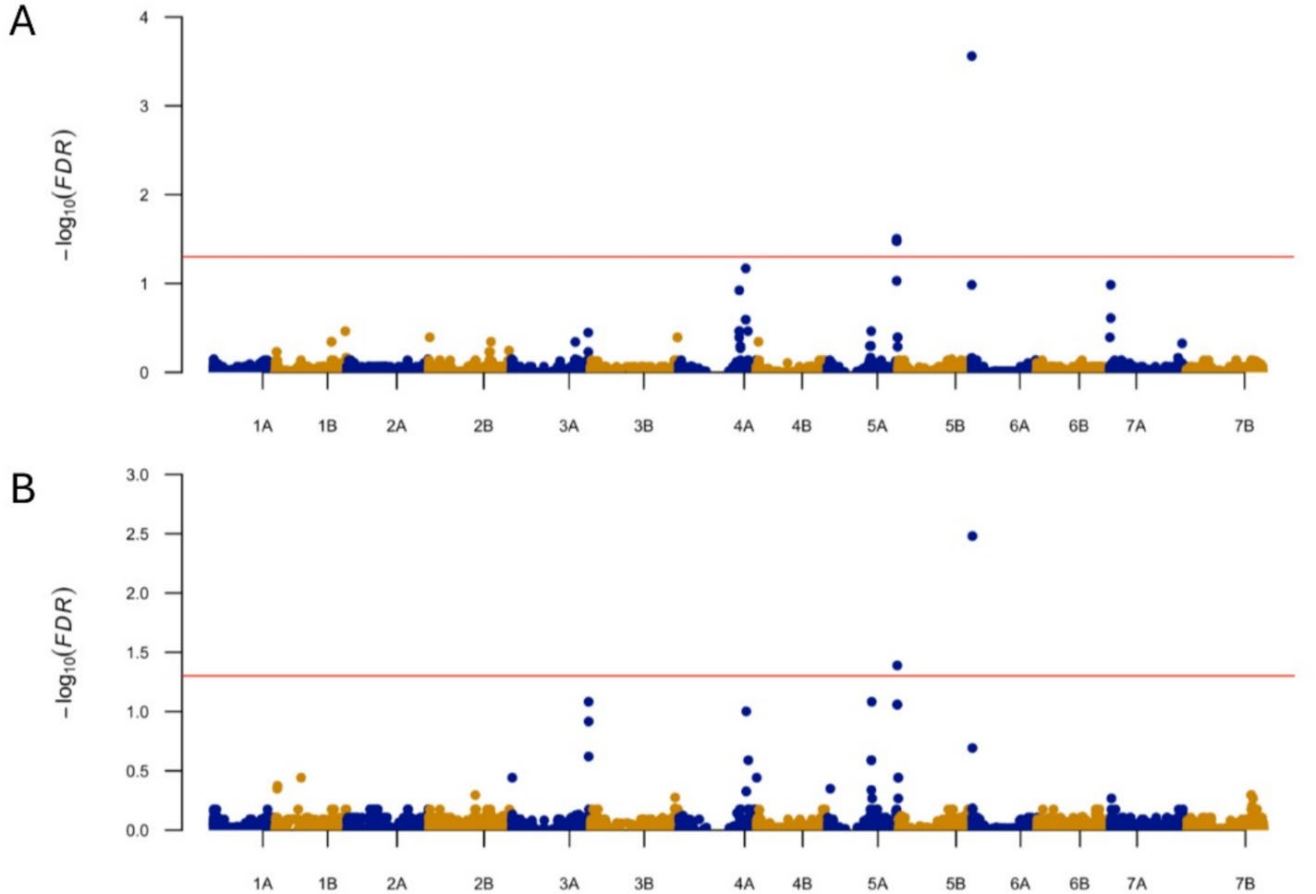
Manhattan plots from the genome-wide association studies (GWAS) of bacterial leaf streak resistance in the Global Durum Panel. The X-axis shows the durum chromosomes and Y-axis shows the values of -Log_10_(*FDR*). A red line indicates the threshold of -Log_10_(*FDR*) = 1.3 (*q* = 0.05) for the detection of significant marker-trait associations. The GWAS was conducted using a model integrated population structure and kinships (PK model in method). **A** results based on average infection-type (AvIT) data and **B** results based on the average percentage of water-soaked area (Av%WS) (color figure online)

Five and two SNP markers were significantly associated with AvIT and Av%WS (Table 3; Fig. 2). A single SNP marker on 6A (*RAC875_c13610_2646*) was identified for both AvIT and Av%WS. This marker had -Log_10_ (*q*) values of 4.0 and 2.5 and explained 6.72% and 5.65% of the phenotypic variation (*R*) for AvIT and Av%WS, respectively. The marker is physically located at the 1,202,829 bp position on 6A of the Svevo v.1 reference genome assembly. The frequency of resistance alleles in the GDP was about 14.3%. Four tightly linked SNP markers on 5A (from 656,981,441 to 657,084,054 bp) were also associated with AvIT having -Log_10_ (*q*) values close to 1.5 and explaining from 4.02% to 4.42% of the phenotypic variation. Only one of these markers was detected for Av%WS with a slightly lower significance value than AvIT (Table 3). The MSD values for BLINK were 0.001368 (AvIT) and 0.00186 (Av%WS) which are greater than the PK model. The MSD values for MLMM were smaller compared to PK model which were 0.0000474 (AvIT) and 0.0000932 (AV%WS). The SNP marker RAC875_c13610_2646 on 6A was still the most significant marker detected for the two models (Table S5). However, SNP markers on other chromosomes including 1A, 1B, 3A, 4A, 5B and 7A were also identified (Table S5).

**Table 3:** Marker-trait association identified for the reaction to bacterial leaf streak in the Global Durum Panel

Trait	Marker	Chr	Position (bp)^a^	Allele	*p* value	q value	*R*^2^ (%)	Effect	MAF^b^ (%)	No. Allele A	No. Allele T
AvIT	*RAC875_c13610_2646*	6A	1,202,829	A/T	2.1061E−8	0.00028	6.72	0.53	14.3	412	69
AvIT	*BS00021688_51*	5A	657,084,054	A/T	4.8118E−6	0.03145	4.42	− 0.45	20.8	381	100
AvIT	*Excalibur_c16470_623*	5A	656,981,441	A/T	1.275E−5	0.03334	4.02	0.42	18.7	90	391
AvIT	*Excalibur_c16470_494*	5A	656,981,570	A/T	1.275E−5	0.03334	4.02	0.42	18.7	90	391
AvIT	*Excalibur_c16470_278*	5A	656,981,786	A/T	1.275E−5	0.03334	4.02	− 0.42	18.7	391	90
Av%WS	*RAC875_c13610_2646*	6A	1,202,829	A/T	2.5322E−7	0.00331	5.65	10.51	14.3	412	69
Av%WS	*BS00021688_51*	5A	657,084,054	A/T	6.251E−6	0.04086	4.31	− 9.65	20.8	381	100

^a^The physical position was based on the reference genome of durum cultivar Svevo v1 (https://plants.ensembl.org/Triticum_turgidum/Info/Index)

^b^MAF = minor allele frequency

### Reaction of two durum biparental populations to BLS

**Fig. 3:**
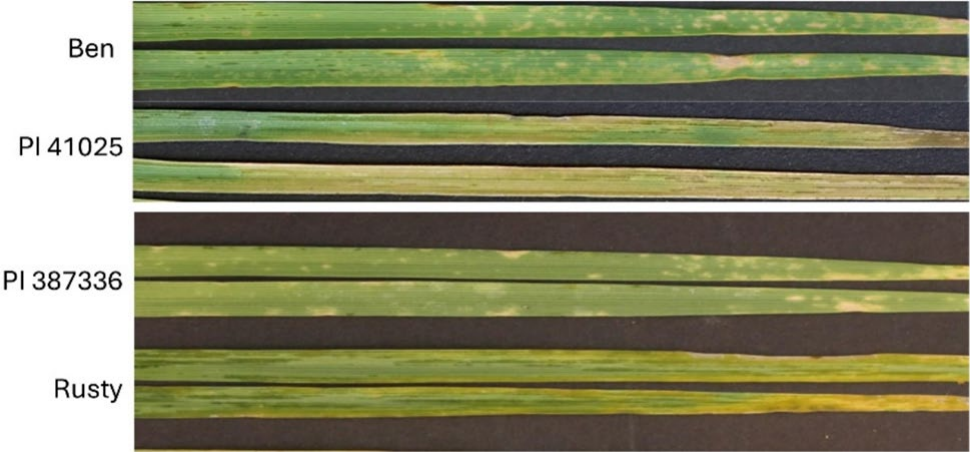
Reaction of the parental lines of two durum recombinant inbred line populations to bacterial leaf streak. The parental lines are Ben and PI 41025 for the BP025 population and PI 387336 and Rusty for RP336 population. The inoculated leaves were photographed 7 days after inoculation. Two representative leaves for each line were included

**Fig. 4:**
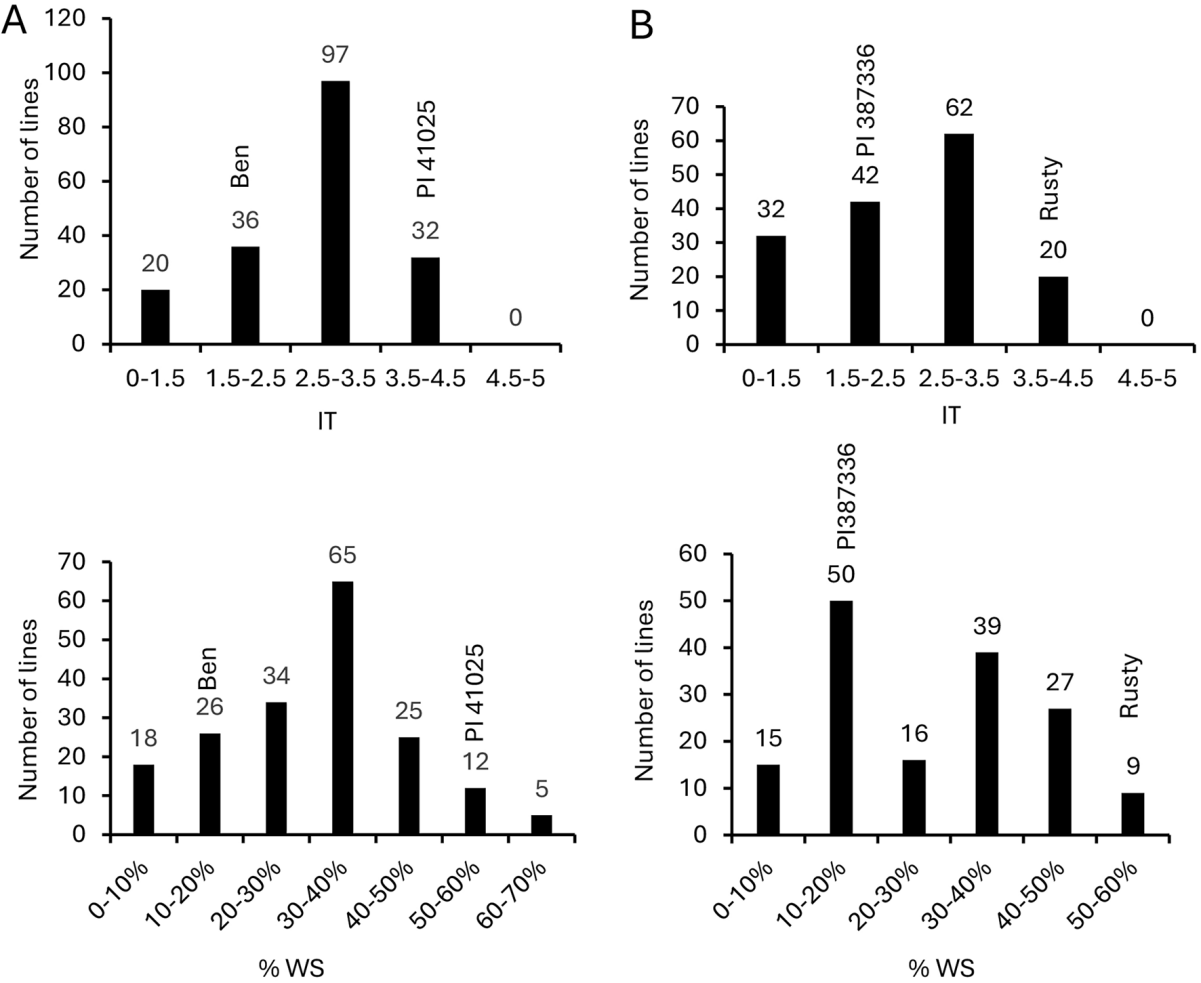
Distribution of the two durum recombinant inbred line populations for reaction to bacterial leaf streak. The *Y*-axis shows the number of recombinant inbred lines and the *X*-axis shows the disease scales used in disease severity scoring. **A** the BP025 population, infection type (IT) on top panel and %WS on the bottom panel; **B** the RP336 population, infection type (IT) on the top panel and %WS on the bottom panel. The number of lines in each column and the disease scores of parental lines are indicated on the top of the columns in each graph

The two parental lines of both biparental populations differed greatly in their reaction to BLS (Fig. 3). Ben and PI 387336 developed small chlorotic lesions with little or no water-soaking symptoms on the leaf indicating resistance. In contrast, PI 41025 and Rusty developed heavy water-soaking symptoms across the entire leaf indicating highly susceptible reactions. Levene’s homogeneity test indicated that the replications of disease evaluations for both populations were homogeneous (*p* = 0.27 for BP025, *p* = 0.41 for RP336). Thus, the average disease data (AvIT and Av%WS) was calculated and used in subsequent analysis. The correlations between the AvIT and Av%WS data were relatively high (*r* = 0.931 for BP025; *r* = 0.83 for RP336). Both RIL populations segregated for reaction to BLS from highly resistant to highly susceptible, as measured by AvIT or Av%WS (Fig. 4). The disease scores of the BP025 population ranged from 1.5 to 4.5 for AvIT and from 4.2 to 73.3% for Av%WS. In the case of the RP336 population, disease scores ranged from 1.1 to 4.2 for AvIT and from 5.7 to 66.2% for Av%WS. Transgressive segregation was observed at varying levels for both populations (Fig. 4).

### QTL identification in BP025 and RP336

**Fig. 5:**
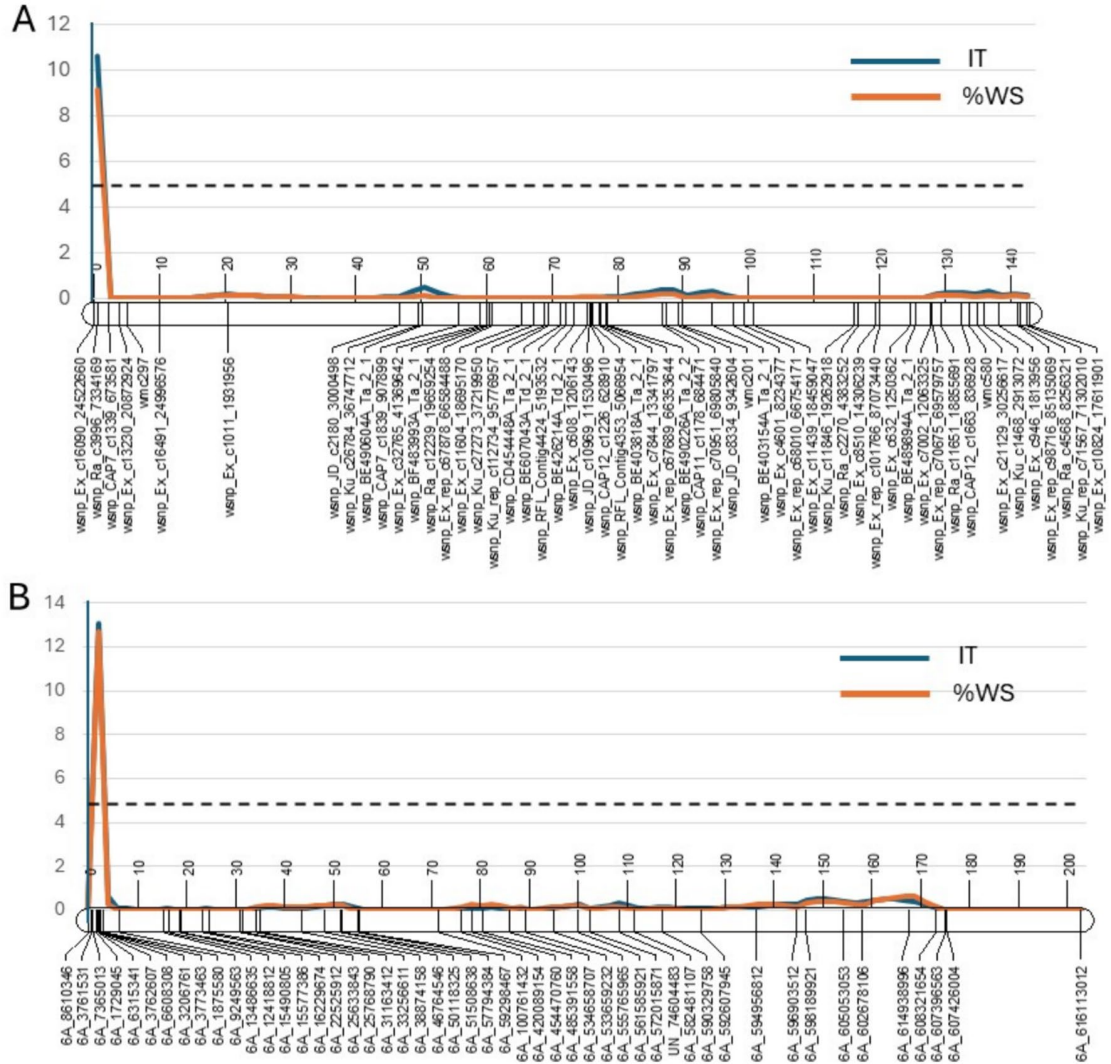
Composite interval mapping of QTL on chromosome 6A associated with reaction to bacterial leaf streak in the two durum wheat populations. **A** The BP025 population; **B** The RP336 population. The chromosome 6A linkage maps were drawn with marker names along the bottom and a centimorgan (cM) scale across the top. The blue line indicates the QTL identified with infection-type data and the orange line indicates the QTL identified with the percentage of water-soaking area data. A dashed line indicates the LOD cutoff (4.7) for QTL detection, which was obtained by performing a permutation test of 1,000 iterations

In the BP025 population, SIM revealed two genomic regions associated with BLS AvIT, one on 6A designated *QBls.zhl-6A(BPIT*) and the other on 5A designated *QBls.zhl-5A(BPIT*) (Table 4). *QBls.zhl-6A*(*BPIT)* was located at the very end of the short arm of chromosome 6A with a LOD value of 9.4 and a *R*^2^ value of 0.21. *QBls.zhl-5A*(*BPLT)* was identified on the long arm of chromosome 5A with a much smaller effect (LOD = 4.1 and *R*^2^ = 0.095). CIM delimited *QBls.zhl-6A(BPIT)*to the first 2.0 cM genetic interval of 6A between markers *wsnp_Ex_c16090* and *wsnp_CAP_C1339* (Fig. 5). The 5A QTL was not significant in CIM. When Av%WS data was used, only one QTL (*QBls.zhl-6A* (*BPWS*)) was identified, which was at the same locus as *QBls.zhl-6A(BPIT)*. This QTL remained significant when using CIM (Fig. 5). The additive effects of all QTL were negative indicating that Ben contributes the resistance alleles of those QTL to reduce disease severity value.

**Table 4:** Quantitative trait locus associated with reaction to bacterial leaf streak based on infection type and percentage of water-soaked area in two durum populations

Population	QTL^a^	Chr	Simple interval mapping					Composite interval mapping		
			Interval (cM)	Flanking markers	LOD^b^	*R*^2 c^	*Add. effect*	Interval	Flanking markers	LOD^b^
*BP025*	*QBls.zhl-6A(BPIT)*	6A	0–14	*Ex_c16090-Ex_C280*	9.4	21.0	− 0.35	0–2	*Ex_c16090-CAP_C1339*	11.0
	*QBls.zhl-5A(BPIT)*	5A	124–136	*IWA2743-IWA7044*	4.1	9.5	− 0.27	–	–	NS^d^
	*QBls.zhl-6A(BPWS)*	6A	0–14	*Ex_c16090-Ex_C280*	*8.1*	18.0	− 5.80	0–2	*Ex_c16090-CAP_C1339*	NS
*RP336*	*QBls.zhl-6A.1(RPIT)*	6A	0–20	*6A_8610346- 6A_15577414*	8.7	23.0	0.44	2–3	*6A_1729034-6A_6608308*	13.0
	*QBls.zhl-6A.2(RPIT)*	6A	148–168	*6A_598189921- 6A_614872222*	3.9	11.0	0.31	–	–	NS
	*QBls.zhl-3A(RPIT)*	3A	14–18	*3A_12287401- 3A_15345505*	3.3	9.2	0.29	–	–	NS
	*QBls.zhl-6A.1(RPWS)*	6A	0–16	*6A_8610346-6A_12418812*	8.2	22.0	7.40	2–3	*6A_1729034-6A_6608308*	12.7
	*QBls.zhl-6A.2(RPWS)*	6A	162–168	*6A_602678106-6A_614872222*	4.1	11.3	5.60	–	–	NS
	*QBls.zhl-3A(RPWS)*	3A	8–24	*3A_1214628-3A_20232548*	4.0	12.0	5.40	–	–	NS

^a^QTL with IT was obtained using infection-type data and WS was obtained using percentage of water-soaking area data.

^b^Permutation test with 1,000 iterations yielded LOD values of 3.2 and 4.7 as the cutoff to identify significant QTL for simple interval mapping and composite interval mapping, respectively.

^c^*R*^2^ value indicates the amount of phenotypic variation explained by the individual QTL for simple interval mapping

^d^NS = not significant

In the RP336 population, three QTL were identified from SIM for AvIT including *QBls.zhl-6A.1(RPIT)* on 6AS, *QBls.zhl-6A.2(RPIT)* on 6AL, and *QBls.zhl-3A(RPIT)* on 3AS (Table 4). Among them, *QBls.zhl-6A.1(RPIT)* had the largest effects with a LOD value of 8.7 and a *R*^*2*^ value of 0.23. This QTL was at a similar position as *QBls.zhl-6A(BPIT)*detected in the BP025 population. The effects of *QBls.zhl-6A.2(RPIT)* and *QBls.zhl-3A(RPIT)* were relatively minor as their LOD values were slightly over the significance threshold. Of these three QTL, only *QBls.zhl-6A.1(RPIT)* was significant when using CIM (Fig. 5). The same three genomic regions were identified with Av%WS data but the genetic interval for each QTL was slightly different (Table 4). Similarly, only *QBls.zhl-6A.1(RPWS)* remained to be significant in CIM (Fig. 5). All QTL had positive additive effects indicating that the resistance alleles were from PI 387336.

## Discussion

A limited number of studies have been conducted to identify sources of BLS resistance in durum wheat. Here, we found that four ND durum cultivars and 20 durum accessions from other regions were resistant to BLS caused by a bacterial strain from ND. These resistant materials could serve as valuable sources of resistance for BLS in durum breeding programs. Using GWAS and QTL analysis, we determined that resistance to BLS in durum is predominantly controlled by a major QTL located on the short arm of chromosome 6A. The associated SNP markers can be converted to KASP markers and effectively utilized in marker-assisted selection to transfer the 6A QTL to elite durum lines. To our knowledge, this is the first study addressing durum resistance to BLS, and our findings provide important information and tools for durum breeding programs. Given the limited sources of resistance available in common wheat, the identified QTL may also be useful for breeding common wheat cultivars with BLS resistance.

The ND durum cultivar showed a range of responses to BLS with most of them being susceptible (IT > 3.0 and %WS > 30%) (Table 1). However, only the durum line Rusty was as susceptible as the susceptible common wheat and triticale checks. This differs from spring wheat cultivars, where most of them are highly susceptible (Sapkota et al. 2018). Four durum cultivars including Alkabo (Elias and Manthey 2007a), Ben, Divide (Elias and Manthey 2007b), and Pierce (Elias et al. 2004), demonstrated comparable levels of resistance to BLS. These cultivars can be utilized by durum growers in ND or surrounding areas to mitigate economic losses associated with the disease. Additionally, they can be incorporated into the North Dakota State University (NDSU) breeding program to enhance BLS resistance in currently popular, susceptible varieties. In contrast, ND Grano (Elias et al. 2021) and ND Riveland (Elias and Manthey 2019), which are relatively new cultivars, were found to be susceptible to BLS. Consequently, these two cultivars are not recommended for use in areas where disease pressure is high.

Our evaluation of 511 world durum accessions revealed that only 20 exhibited high levels of resistance, highlighting the scarcity of BLS resistance in durum wheat. It is crucial to perform disease screenings on a broader range of germplasm, including cultivated and wild emmer wheat such as the study by Khan et al. (2025) who reported 14 resistant lines among 508 cultivated emmer accessions. Nevertheless, the 20 resistant durum lines that we identified can serve as primary materials for breeding programs aimed at improving cultivar resistance to BLS. Since most of the resistant accessions are varieties or breeding lines, no pre-breeding steps are necessary to enhance their agronomic traits. Among these resistant accessions, nine originated from France and five from the International Center for Agricultural Research in Dry Areas (ICARDA). It remains to be determined whether the resistant accessions from the same country share identical resistance genes. Genetic analysis using developed biparental populations would be a logical next step to determine their genetic relationships concerning resistance.

Through GWAS and biparental mapping, we identified a genomic region on chromosome 6A associated with BLS resistance. Based on genetic positions, it is likely that the QTL identified from the GDP and the two durum biparental populations are the same. The identification of this QTL using different methodologies and from multiple sources suggests its robustness and significance. Although no QTL mapping has been reported for durum, studies on common wheat have revealed numerous BLS resistance QTL on various chromosomes including 1A, 1B, 2A, 2B, 3A, 3B, 4A, 5A, 5B, 5D, 6B, 6D, 7A, 7B, and 7D (reviewed in Ledman et al. 2023; Acharya et al. 2024). However, no QTL has been identified on chromosome 6A. Even in emmer wheat germplasm, Khan et al. (2025) did not detect any marker-trait association (MTA) on chromosome 6A. It is possible that the QTL identified on 6A is specific to durum wheat and is absent in common wheat and other wheat germplasm.

The only significant marker identified on chromosome 6A in the GWAS was *RAC875_c13610_2646*, located at the 1,202,829 bp position of the Svevo 6A chromosome assembly. In the BP025 population, the QTL peak was underscored by three co-segregated SNP markers at the 0.0 cM position of the 6A linkage map: *wsnp_Ex_c16090_24522660* (at 6A: 1,649,488 bp), *wsnp_Ku_c26585_36553202* (at 6B: 2,901,044 bp, no hit on 6A), and *wsnp_Ex_rep_c67563_66193104* (at 6A: 1,197,947 bp). It is noteworthy that *wsnp_Ex_rep_c67563_66193104* is physically very close to *RAC875_c13610_2646* identified in the GWAS. Both markers were developed from the same gene, *TRITD6Av1G000770*, which encodes an E3 ubiquitin-protein ligase. E3 ubiquitin-protein ligases play important roles in the plant protein ubiquitination process and have been implicated in plant defense responses against biotic and abiotic stresses (Dulpan et al., 2014; Fu et al. 2022; Su et al. 2024). Whether this gene confers BLS resistance in durum or not remains to be tested.

The genetic interval of *QBls.zhl-6A*(*RPIT)* identified in RP336 is not located at the end of chromosome 6A. We observed that the genetic order of the markers in that region does not align well with their physical order (Fig. 5, Liu et al. 2020). This discrepancy may indicate chromosome rearrangements in this population. We also assessed the markers for missing values and found that up to 48% of the data for these markers were missing. As a result, the genetic order for these markers may not be reliable. However, we identified the most significant marker for the QTL (*QBls.zhl-6A* (*RPIT*)) as *6A_1875580* (located at 6A:1,875,580), which is in close proximity to the markers identified in the GWAS and the BP025 population. This further suggests that the same QTL is responsible for BLS resistance in the materials we evaluated.

GWAS identified several markers on chromosome 5A that are associated with BLS resistance, located approximately at the 657 Mb position (see Table 3). A minor QTL (*QBls.zhl- 5A(BPIT)*) was also located on 5A between markers *IWA2743* and *IWA7044* in the BP025 population using SIM analysis. The approximate physical positions of the two flanking markers in the Svevo genome range from 548 to 571 Mb, which is relatively close to the 657 Mb position. Additionally, Acharya et al. (2024) discovered a QTL on 5A for BLS resistance in a common wheat population, with the peak marker situated at the 639 Mb position in the Svevo genome. These QTLs likely correspond to those conferring BLS resistance in both durum and common wheat. SIM analysis also revealed another QTL (*QBls.zhl-3A(RPIT)* and *QBls.zhl-3A (RPWS*) in the RP336 population at the end of the 3A chromosome. A 3A QTL was identified by Ramakrishnan et al. (2019) in a winter wheat panel, but it was located more centrally on the 3A chromosome. The 3A QTL identified in our study may be a homolog of the 3B QTL discovered by Acharya et al. (2024). Both the 5A and 3A QTLs exhibited much smaller effects compared to the 6A QTL and were not significantly detected using CIM. While these QTLs could indeed be legitimate, they may not meet the cutoff criteria due to a low number of markers or the small size of the population studied.

In conclusion, we identified a set of durum wheat lines exhibiting resistance to BLS through screening major ND durum cultivars and a collection of global durum accessions. Through GWAS and biparental population mapping, we identified a major QTL on 6A that is associated with BLS resistance. The identified resistant lines and the markers associated with the major QTL should prove valuable for breeding efforts aimed at improving BLS resistance in both durum and common wheat.

## Supplementary Information

Below is the link to the electronic supplementary material.

Supplementary file1 (DOCX 423 KB)

Supplementary file2 (XLSX 66 KB)

Supplementary file3 (DOCX 16 KB)

Supplementary file4 (DOCX 16 KB)

Supplementary file5 (XLSX 81 KB)

Supplementary file6 (XLSX 12 KB)
