# Supplementary material for: Genome-wide association and biparental mapping revealed a major quantitative trait locus associated with seedling resistance to bacterial leaf streak in durum
Source: Theor Appl Genet. 2025 Dec 19;139(1):10. doi: 10.1007/s00122-025-05111-7 (PMC12717212; doi:10.1007/s00122-025-05111-7)
Supplement: Supplementary file 1 — Supplementary file1 (DOCX 423 KB) [file 122_2025_5111_MOESM1_ESM.docx]

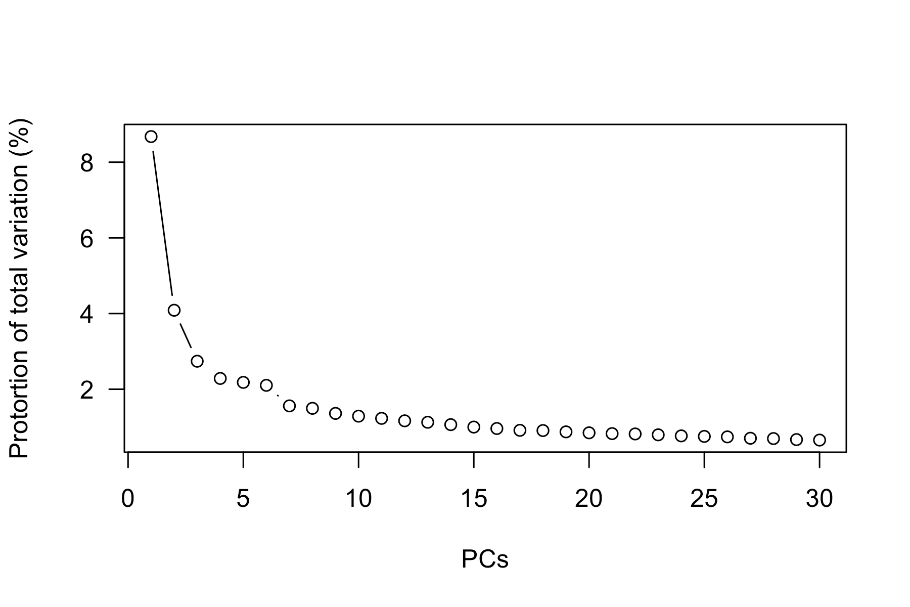


**Figure S1.** Scree plot of principal components (PC) from the Global Durum Panel genotypic data. The X-axis shows the number of PC and Y-axis shows the proportion of total variation (%) for each PC.

**
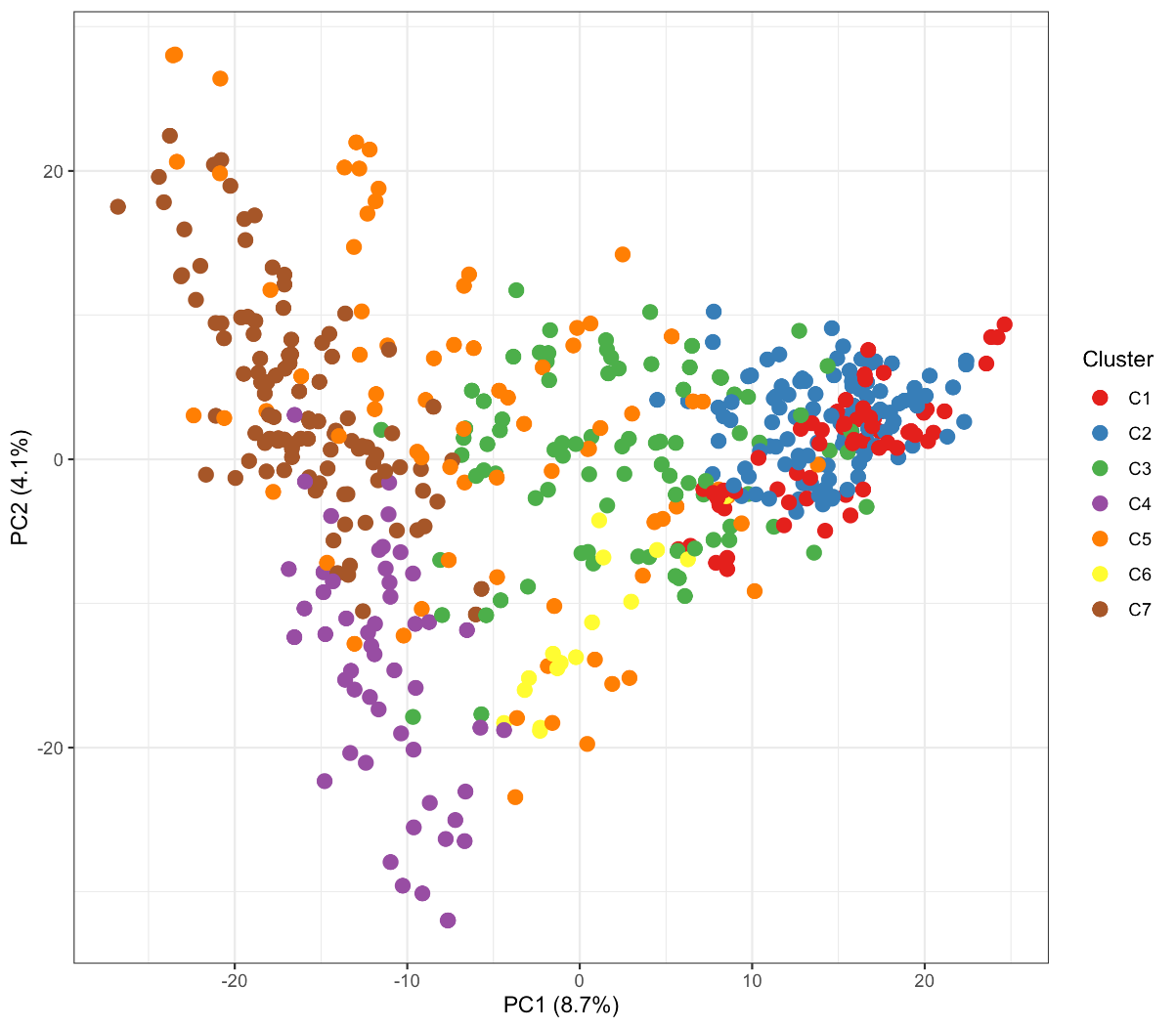
**

**Figure S2.** Scatter plot of the 511 durum accessions according to the first two principal component. PC1 and PC2 with their effects are shown on the X-axis and Y axis, respectively. The clusters described in results and Table S4 are indicated with different colors.
