# Supplementary material for: Genome-wide association and biparental mapping revealed a major quantitative trait locus associated with seedling resistance to bacterial leaf streak in durum
Source: Theor Appl Genet. 2025 Dec 19;139(1):10. doi: 10.1007/s00122-025-05111-7 (PMC12717212; doi:10.1007/s00122-025-05111-7)
Supplement: Supplementary file 3 — Supplementary file3 (DOCX 16 KB) [file 122_2025_5111_MOESM3_ESM.docx]

**Table S2.** Analysis of variance (ANOVA) for bacterial leaf streak infection type (IT) and percentage water-soaked area (%WS) in durum cultivars screened in the greenhouse

| Trait | Source of variation | DF | Sum of squares | | Mean square | | *F* value | *P* > F |
| --- | --- | --- | --- | --- | --- | --- | --- | --- |
| IT | Genotype | 17 | 47.63 |  | 2.80 |  | 21.98 | < 0.0001 |
|  | Error | 30 | 3.36 |  | 0.13 |  |  |  |
|  | Corrected total | 47 | 51.45 |  |  |  |  |  |
| %WS | Genotype | 17 | 16434.19 |  | 966.72 |  | 14.53 | < 0.0001 |
|  | Error | 30 | 1996.13 |  | 66.55 |  |  |  |
|  | Corrected total | 47 | 18430.80 |  |  |  |  |  |
