# Supplementary material for: Genome-wide association and biparental mapping revealed a major quantitative trait locus associated with seedling resistance to bacterial leaf streak in durum
Source: Theor Appl Genet. 2025 Dec 19;139(1):10. doi: 10.1007/s00122-025-05111-7 (PMC12717212; doi:10.1007/s00122-025-05111-7)
Supplement: Supplementary file 4 — Supplementary file4 (DOCX 16 KB) [file 122_2025_5111_MOESM4_ESM.docx]

**Table S3.** ANOVA analysis for reaction of the Global Durum wheat Panel (GDP) to bacterial leaf streak based on infection type and percentage of water-soaked area in the greenhouse.

| Trait | Source of variations | DF | Sum of squares | | Mean square | | *F* value | *P*> F |
| --- | --- | --- | --- | --- | --- | --- | --- | --- |
| IT | Genotype | 490 | 778.78 |  | 1.59 |  | 10.31 | < 0.0001 |
|  | Error | 1383 | 213.16 |  | 0.15 |  |  |  |
|  | Corrected total | 1873 | 991.94 |  |  |  |  |  |
| %WS | Genotype | 490 | 316336.64 |  | 645.59 |  | 5.88 | < 0.0001 |
|  | Error | 1383 | 151832.00 |  | 109.78 |  |  |  |
|  | Corrected total | 1873 | 468168.64 |  |  |  |  |  |
